# The mechanics of abalone crawling on sharp objects without injury

**DOI:** 10.1038/s41598-019-40505-w

**Published:** 2019-03-07

**Authors:** Yun Zhang, Shanpeng Li, Pingcheng Zuo, Jiaxin Ji, Jianlin Liu

**Affiliations:** 1 Department of Engineering Mechanics, College of Pipeline and Civil Engineering, China University of Petroleum (East China), Qingdao, 266580 China; 2 College of Mechanical and Electronic Engineering, China University of Petroleum (East China), Qingdao, 266580 China

## Abstract

Despite the soft appearance of their feet, abalones can crawl quickly on sharp objects. Tests using rough substrates aligned with blades or posts found that the animal has two adaptations to guarantee its safety on these surfaces. Mechanical compression tests showed that the abalone foot muscle is inherently robust and can resist penetration by sharp objects. A finite element simulation indicated that to avoid being pierced, abalone controls the shape of its foot to wrap it around sharp objects, thereby greatly reducing the stress concentration. These analyses may aid the engineering of new materials and devices for fields including soft robotics and aircraft.

## Introduction

Over millions of years, many creatures have evolved amazing adaptions to their environments. An example in plants is the ultrahydrophobicity of lotus, rice, and *Alchemilla* leaves, which arises from the micro/nano surface structures and keeps rain drops and dust off the leaves efficiently^1–3^. In the animal kingdom, cockroaches can climb vertical walls and run across ceilings quickly^4–6^, but can also bear considerable compressive forces by deforming their bodies so that their height is reduced by 40%–60%^7^. Mosquitos, which are numerous in humid and rainy areas, have a remarkably strong, light exoskeleton that helps them survive the impact of raindrops of more than 50 times their body weight^8^. Namib Desert beetles meet their need for water in their extremely arid environment by collecting fog via elaborate microstructures on their carapaces^9,10^. Some aquatic creatures, such as water striders, can run and jump on the surface of water because of the double-levelled microstructures on their legs and their ability to adjust the flexibility of their legs automatically in response to the deformed water–air interface^11–13^. Similarly, gecko feet also have numerous setae-based nanofibres on their surface^14^, which produce strong van der Waals forces that allow geckos to walk up walls^15–18^.

Although the adhesion force of geckos’ feet is greatly decreased underwater^19^, many marine organisms, such as mussels^20^ and barnacles^21,22^, adhere tightly to the surfaces of ships, rocks, and iron platforms. Abalone, a popular edible marine mollusc, can generate a huge adhesion force underwater^23,24^. Li *et al*. recently measured the adhesion force of abalone in the normal and tangential directions, and found that the forces are greatly affected by the wetting property and morphology of the substrate surface^23^. Abalones are generally observed when they are static and attached to rock surfaces, giving the inaccurate impression that they do not move readily. However, they are not sessile, and can occasionally crawl across rocks using their feet^25,26^. Moreover, Donovan *et al*.^27^ reported that abalone adopts an elegant strategy to reduce the energy cost of fast movement, and can reach a velocity of 86.73 cm/min. Abalones feed mainly on seaweed, which they usually seek after dusk^28–30^ to avoid predators. They escape from predators by crawling swiftly on the seabed. This raises the question of how the soft, tender abalone foot is not injured by sharp rocks, shells, or coral. In addition, abalones adhere firmly to sharp substrates when scared, without sustaining damage. These astonishing behaviours are of considerable interest, and the many mechanical properties of abalone deserve to be explored.

In this study, we examine the mechanisms that abalones use to avoid injury by sharp objects. Substrates aligned with different sharp blades or posts are tested to investigate the mollusc’s motion. The force that the abalone foot can bear is also tested. Finite element method (FEM) simulations are conducted to demonstrate that the foot can modulate its configuration to ensure its security on sharp surfaces.

## Results and Discussion

**Figure 1:**
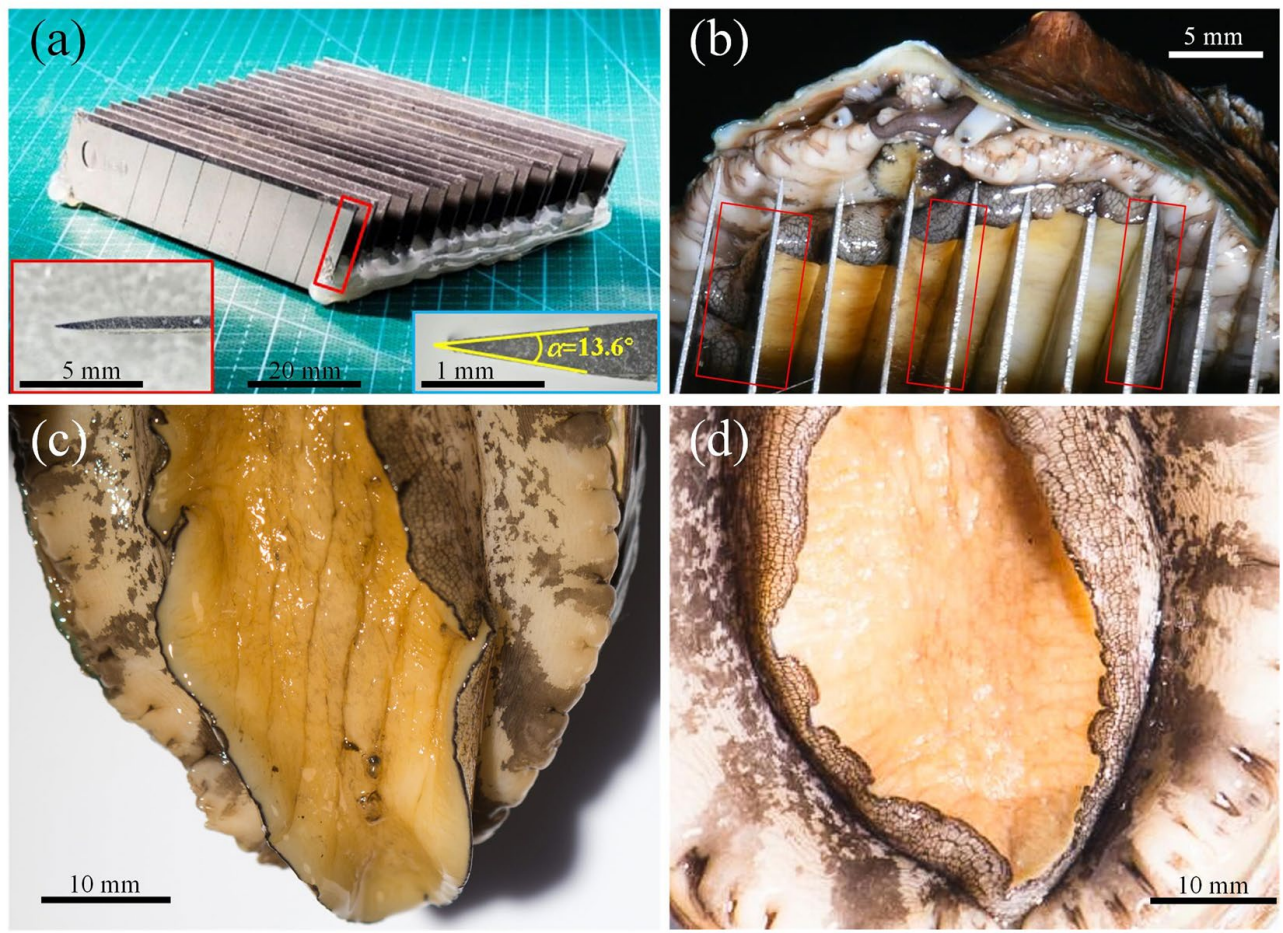
Abalone crawling on sharp blades. (**a**) Parallel blades aligned on the substrate. The insets show enlargements of the side view of the single blade indicated by the red box. The right inset shows the included angle as *α* = 13.6°, and the top surface of the blade is a rectangular area. (**b**) Bottom view of an abalone foot on the blades, which indented the sole by around 3 mm. (**c**) After removal from the blades, grooves about 2.13 mm deep remained on the sole. (**d**) Appearance of the abalone foot after several minutes. The grooves had disappeared.

**Figure 2:**
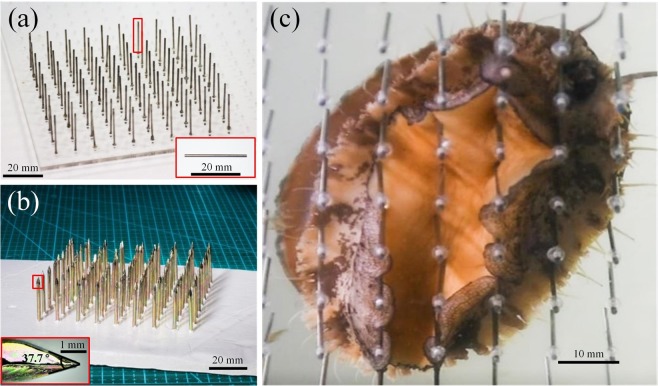
Abalone crawling on cylindrical and pointed posts. (**a**) The array of cylindrical posts; the inset shows the shape of a single post. (**b**) The array of pointed posts; the inset shows the included angle at the point, *β* = 37.7°. (**c**) Bottom view of an abalone on the cylindrical posts.

The abalone was tested underwater on sharp surfaces consisting of parallel blades (Fig. 1), and periodic arrays of cylindrical or pointed stainless steel posts (Fig. 2). The abalone moved rapidly on all three surfaces with a velocity of around *v* = 39.1 ± 3.17 cm/min, which was twice that on smooth surfaces (about 17.8 ± 2.81 cm/min). Figure 1b shows a possible strategy of the abalone muscle wrapping around the blade, and then adhering to its sides. The contact position of the abalone on the blade indicates that the blade edge was clamped by the abalone, with the blade indenting the sole by around 3 mm. After being pulled from the blades, some grooves (around 2.13 mm deep) remained on the surface of the sole (Fig. 1c), but they disappeared after several minutes without leaving any injury (Fig. 1d). Similarly, a bottom view of the abalone foot in contact with the cylindrical posts (Fig. 2c) shows the muscle encircling the posts and adhering to their side surfaces. The posts indented beyond the line of the sole by approximately 2.33 mm and also left no injury on the abalone foot after the animal’s removal from either of the post types.

**Figure 3:**
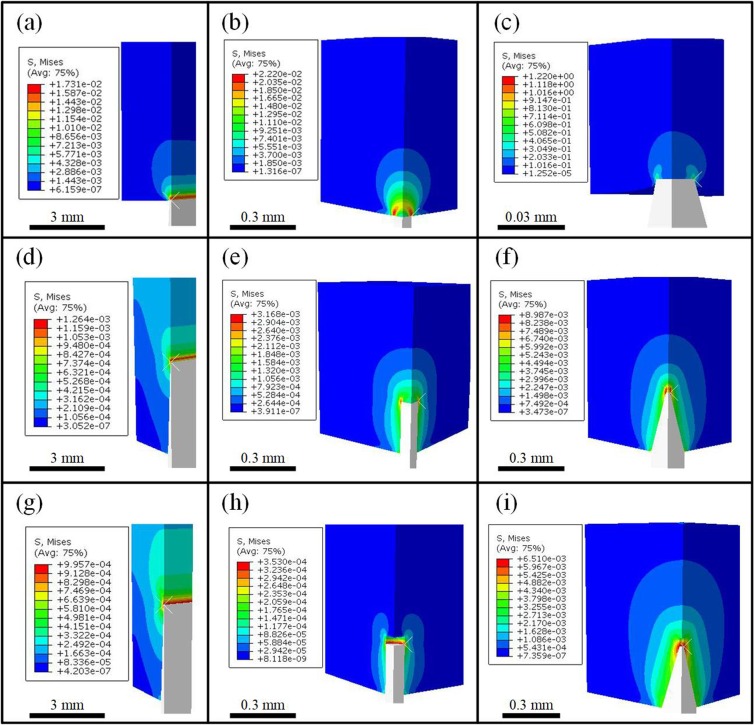
Von Mises stress distributions of the abalone on sharp surfaces simulated by FEM. Distributions from when the abalone is put on the (**a**) blades, (**b**) cylindrical posts, and (**c**) pointed posts; when the abalone foot fully wraps around the (**d**) blades, (**e**) cylindrical posts, and (**f**) pointed posts (i.e., it directly touches the top of each object); and when the abalone foot adheres to the sides of the (**g**) blades, (**h**) cylindrical posts, and (**i**) pointed posts (leaving a gap between the foot and each object’s top).

The following analysis outlines the mechanics of the abalone’s avoidance of injury on these sharp surfaces. As soon as the abalone is placed on these sharp surfaces it has not had time to respond and modulate its posture, and thus the contact surface is approximately planar. Under this assumption, the pressure on the contact area of the foot can be estimated as

1 $${p}_{i}=\frac{{G}_{a}-{F}_{a}}{{n}_{i}{A}_{i}},$$

where *G*_*a*_ is the gravitational force of the abalone body, *F*_*a*_ is its buoyancy, *n*_*i*_ is the number of objects in contact with the abalone foot, and *A*_*i*_ is the real contact area of the foot with a single object. Subscript *i* = 1, 2, or 3 is adopted to indicate blades, cylindrical posts, or pointed posts, respectively. Although the blades or pointed posts are sharp, there is in practice always a finite contact surface between each of them and the abalone foot, which is either rectangular or circular. For blades of width *b* and length *l*, this area, *A*_1_, is expressed as *bl* (Fig. 1a). For the cylindrical and pointed posts, the areas *A*_2_ and *A*_3_ are given as π*r*_*i*_^2^, where *r*_*i*_ is the contact radius between the abalone and the solid. In addition, we also calculated a more accurate estimate via an FEM simulation to identify the failure mechanism of the foot muscle’s structure and make some comparisons (Fig. 3). Therefore, without loss of generality, the abalone foot material is modelled as a linearly elastic Hookean material. Figure 3a–c show the non-uniform distributions of the von Mises stress, *σ*_eq*i*_ (*i* = 1, 2, 3) on the foot, and *σ*_eq*i*_ is greatest on the periphery of the contact area. The maximum vertical stresses, taken here as the von Mises stress, calculated by FEM are different from those predicted by Eq. (1), but they are of a similar order of magnitude (Table 1). This is because the simplified model in Eq. (1) does not consider stress concentration, whereas the FEM simulation is based on a more rigorous three-dimensional model.

**Table 1:** Pressure and stress values obtained from the experiments and FEM simulations.

Pressure/Stress (Pa)	[*p*_*i*_]	*p*_*i*_	*σ*_eq *i*_	${{\boldsymbol{\sigma }}}_{{\bf{eq}}{\boldsymbol{i}}}^{{\bf{I}}}$	${{\boldsymbol{\sigma }}}_{{\bf{eq}}{\boldsymbol{i}}}^{{\bf{II}}}$
Blade	3.578 × 10^7^	1.61 × 10^4^	1.73 × 10^4^	1264	996
Cylindrical posts	2.916 × 10^7^	2.37 × 10^4^	2.22 × 10^4^	3168	353
Pointed posts	2.525 × 10^8^	3.68 × 10^5^	1.22 × 10^6^	8987	6510

**Figure 4:**
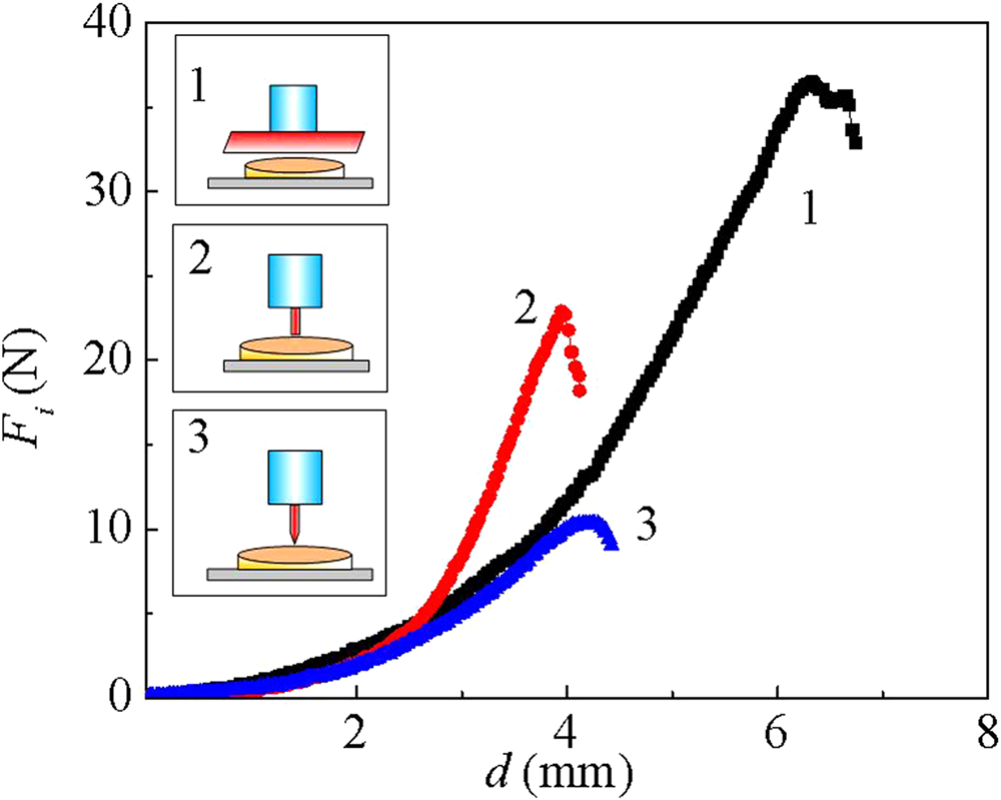
Force–displacement curves of the abalone foot. The curves were exposed to a blade (line 1), cylindrical posts (line 2), and pointed posts (line 3). Schematics of the test corresponding to the line numbers are shown in the three insets.

The first possible reason that abalone can crawl safely on sharp surfaces is that the maximum equivalent stress of the muscle is within the limit stress, namely, the foot is sufficiently robust to resist the external forces. A series of ultimate strength tests on the foot in contact with each of the three sharp surfaces generated the force–displacement curves in Fig. 4, which show two stages. In the first stage, the force increases slowly because the contact of the sharp surfaces with the soft muscle of the abalone is accompanied by a large deformation of the muscle. In the second stage, the contact attains a stable state, and the muscle still has sufficient capability to resist the external force. As the force gradually increases further to its peak of *F*_max*i*_, the sole’s surface is finally broken and the pressure attains the tolerant strength, [*p*_*i*_]. The displacements at the critical peaks for each of the three sharp surfaces (*i* = 1, 2, 3) are 6.33, 4.19, and 4.53 mm, respectively. The tolerant strength is defined as

2 $$[{p}_{i}]=\frac{{F}_{\max i}}{{A}_{i}}.$$

The values of [*p*_*i*_] are derived from these force–displacement curves, and are far greater than the values of the maximum stresses for the three substrates, i.e.

3 $${p}_{i} < [{p}_{i}]\,{\rm{a}}{\rm{n}}{\rm{d}}\,{\sigma }_{{\rm{e}}{\rm{q}}i} < [{p}_{i}].$$

This relation indicates that the abalone is safe on these sharp surfaces as its sole does not collapse.

When the abalone crawls, its acceleration has dynamic effects and increases the stress generated by the contact. The short period of acceleration is several seconds, and the abalone reaches a velocity, *v*, of 39.1 cm/min. The acceleration and dynamic force can be estimated as ~0.1 cm s^−2^ and ~10^−4^ N, respectively. Furthermore, undersea currents also affect the stress concentration. Consequently, the strength condition in Eq. (3) may not be sufficient to guarantee the safety of the abalone in practice, and there are likely to be other strategies that abalones use.

Experiments have found that abalones wrap their highly flexible feet around sharp objects on substrates. Abalones use the antennae on their heads to obtain information about the morphology of a substrate before they traverse it in order to judge whether the substrate is dangerous^31,32^. Once the animal notices that these sharp objects are potentially dangerous, it protects itself by adhering to the side surface of the objects. However, it is not known whether the tip of a sharp object touches the abalone foot directly. Thus, there are two possible contact states between the abalone and a sharp object. In contact state I, the abalone foot directly touches the solid at the tip of the sharp object, fully wrapping around it. In contact state II, the abalone does not touch the tip of the sharp object, separating its foot slightly (0.3 mm in the simulation) from the object. In this case, the foot still adheres to the side surfaces of the object.

For adhesion to the three substrates tested here, the stress fields for contact states I and II, ${\sigma }_{{\rm{eq}}i}^{{\rm{I}}}$ and ${\sigma }_{{\rm{eq}}i}^{{\rm{II}}}$ (*i* = 1, 2, 3), respectively, were quantitatively calculated by FEM (Fig. 3). The data in Table 1 show that the stress values for contact state I are greater than those for contact state II, especially for the cylindrical posts. This is because the muscle avoided the sharp edges of the blades or posts, which reduced the stress concentration. In addition, the FEM values of the von Mises stress for contact states I and II are much smaller than the corresponding values in the initial contact states, namely, ${\sigma }_{{\rm{eq}}i}^{{\rm{II}}}$ < ${\sigma }_{{\rm{eq}}i}^{{\rm{I}}}$ < ${\sigma }_{{\rm{eq}}i}$. The percentage reduction in von Mises stress for contact states I and II, ${\varphi }_{i}^{{\rm{I}}}$ and ${\varphi }_{i}^{{\rm{II}}}$, respectively, are defined as

4 $${\varphi }_{i}^{{\rm{I}}}=\frac{{\sigma }_{{\rm{eq}}i}^{{\rm{I}}}-{\sigma }_{{\rm{eq}}i}}{{\sigma }_{{\rm{eq}}i}}\,{\rm{and}}\,{\varphi }_{i}^{{\rm{II}}}=\frac{{\sigma }_{{\rm{eq}}i}^{{\rm{II}}}-{\sigma }_{{\rm{eq}}i}}{{\sigma }_{{\rm{eq}}i}}$$

The values are calculated as ${\varphi }_{i}^{{\rm{I}}}$ = 92.69%, 85.73%, and 99.26%; and ${\varphi }_{i}^{{\rm{I}}{\rm{I}}}$ = 94.24%, 98.41%, and 99.47% for *i* = 1, 2 and 3, respectively. This wrapping strategy appears to decrease the stress concentration greatly compared with the initial contact state, and thus allows the abalone to crawl on a broader range of sharp surfaces.

In conclusion, we examined the special ability of the abalone to protect itself when crawling on sharp substrates. We found that the muscle of the abalone’s foot is sufficiently strong to bear the pressure caused by sharp objects. In addition, the creature can deform its foot to avoid being pierced by sharp objects. To reduce the stress concentration, the abalone encircles the dangerous area of a sharp object using its flexible foot, thereby reducing the stress by more than 90%. We hope these analyses will help engineers to develop new materials and devices in fields such as soft robotics and aircraft.

## Methods

A camera (D720, Nikon, 4000 × 6000 dpi) was used to record the crawling and adhesion of abalone on sharp substrates. The shapes of the substrate surfaces, as observed by an extended depth-of-field microscope (LY–WN–YH3D, Cheng Du Li Yang Precision Machinery Co. Ltd.), were used to calculate the vertical stress of the abalone foot at the moment of contact with the substrate. Commercial software (ABAQUS 6.14, Dassault Systèmes) accurately computed the equivalent stress when the abalone was adhered to the substrate.

### Abalone and sharp objects

The abalones considered here were *Haliotis discus hannai*, an edible variety from the coastal area of Qingdao City, China. Ten 2.5-year-old cultivated abalone were tested. The mean body length was 6.8 ± 0.7 cm, and the body weights ranged from 48 to 52 g. Abalones were kept in individual transparent aquariums (80 × 60 × 100 cm^3^), and were fed kelp every three days. The tanks were equipped with a chiller and a filtration system, and were maintained at 19 to 20 °C.

Three substrates were created from sharp objects: blades (Fig. 1a), cylindrical steel posts (Fig. 2a), and pointed posts (Fig. 2b). The blades were 100 mm long, 18 mm wide, and 0.5 mm thick, and were placed 3.6 mm apart. The included angle of each blade (the angle between the two side surfaces) was *α* = 13.6°, and although sharp, the edge was regarded as a rectangle of width *b* = 0.034 mm and length *l* = 100 mm. The cylindrical steel posts were of height *h*_2_ = 25 mm and radius *r*_2_ = 0.5 mm. The pointed posts were of height *h*_3_ = 30 mm, conical angle *β*  = 37.7°, and tip radius *r*_3_ = 0.22 mm. Both types of posts were placed 10 mm apart. The blades and posts were washed and sterilized before being fixed at the bottom of the tank.

### Tolerance strength testing

The soles of two abalone were compressed with a universal testing machine (UTM–1432, Cheng De Jin Jian Testing Instrument Co. Ltd.). After immersion in 5% MgCl_2_ in seawater^33,34^, the abalone foot was first cut off along the attachment muscle, and placed on the platform of the material testing machine. Substrates with the three types of sharp object were then fixed to the punch of the testing machine, which moved them downward into the abalone. The loading velocity was set as 20 mm/min, which ensured the experiment was in a quasi-static state^35,36^. Ten different positions on the abalone foot surfaces were measured. The experiments were carried out at room temperature, around 24 °C. Force–displacement curves were recorded by the testing machine.

### Young’s modulus and Poisson’s ratio testing

Five cuboid (30 × 8 × 6 mm) samples, cut from the sole of an anaesthetized abalone, were clamped in the universal testing machine (UTM–1432). The loading velocity was kept at 20 mm/min, and stress–strain curves in the longitudinal direction were obtained. The Young’s modulus was measured as *E*_*a*_ = 0.994 ± 0.32 MPa, and the Poisson’s ratio was *ν* = 0.16 ± 0.09.

### FEM simulation

ABAQUS 6.14 simulated the contact between the abalone foot and the sharp substrates; only one quarter of the configuration was calculated due to its symmetry. The blade, both posts and the abalone were considered as isotropic materials. The Young’s modulus of the steel used in the blades and posts was 21,000 MPa, which was much greater than that of the abalone. The simulation used the element C3D8R. The contact approach between the substrate and the abalone was set as hard contact. The mesh numbers for modelling the abalone on the blade were 2400 for the blade, 750,000 for the foot before contact, and 68,628 after full adhesion. Those for the cylindrical posts were 4800 for the cylinder, 680,000 for the foot before contact, and 655,312 after full adhesion. Those for the pointed posts were 3406 for the post, 364,000 for the foot before contact, and 196,640 after full adhesion.
